# Liquid clues: tracking early-stage breast cancer with ctDNA - a mini review

**DOI:** 10.3389/fonc.2025.1634859

**Published:** 2025-08-27

**Authors:** Saumya Easaw, Johann Hsu, Nury Steuerwald, Arielle L. Heeke

**Affiliations:** ^1^ Department of Solid Tumor Oncology and Investigational Therapeutics, Levine Cancer Institute, Atrium Health, Charlotte, NC, United States; ^2^ Molecular Biology Core Laboratory, Levine Cancer Institute, Atrium Health, Charlotte, NC, United States

**Keywords:** ctDNA, early breast cancer (EBC), MRD - minimal residual disease, personalized medicine, recurrence risk

## Abstract

Circulating tumor DNA (ctDNA) is a testing modality that has several potential applications in the management of non-metastatic breast cancer. In this mini review, we discuss its role in early detection, treatment response assessment, and surveillance for minimal residual disease (MRD). We also review technological advances that may increase assay sensitivity. Finally, we highlight key prospective therapeutic intervention trials that evaluate the incorporation of ctDNA into clinical management of early breast cancer.

## Introduction

**Figure 1:**
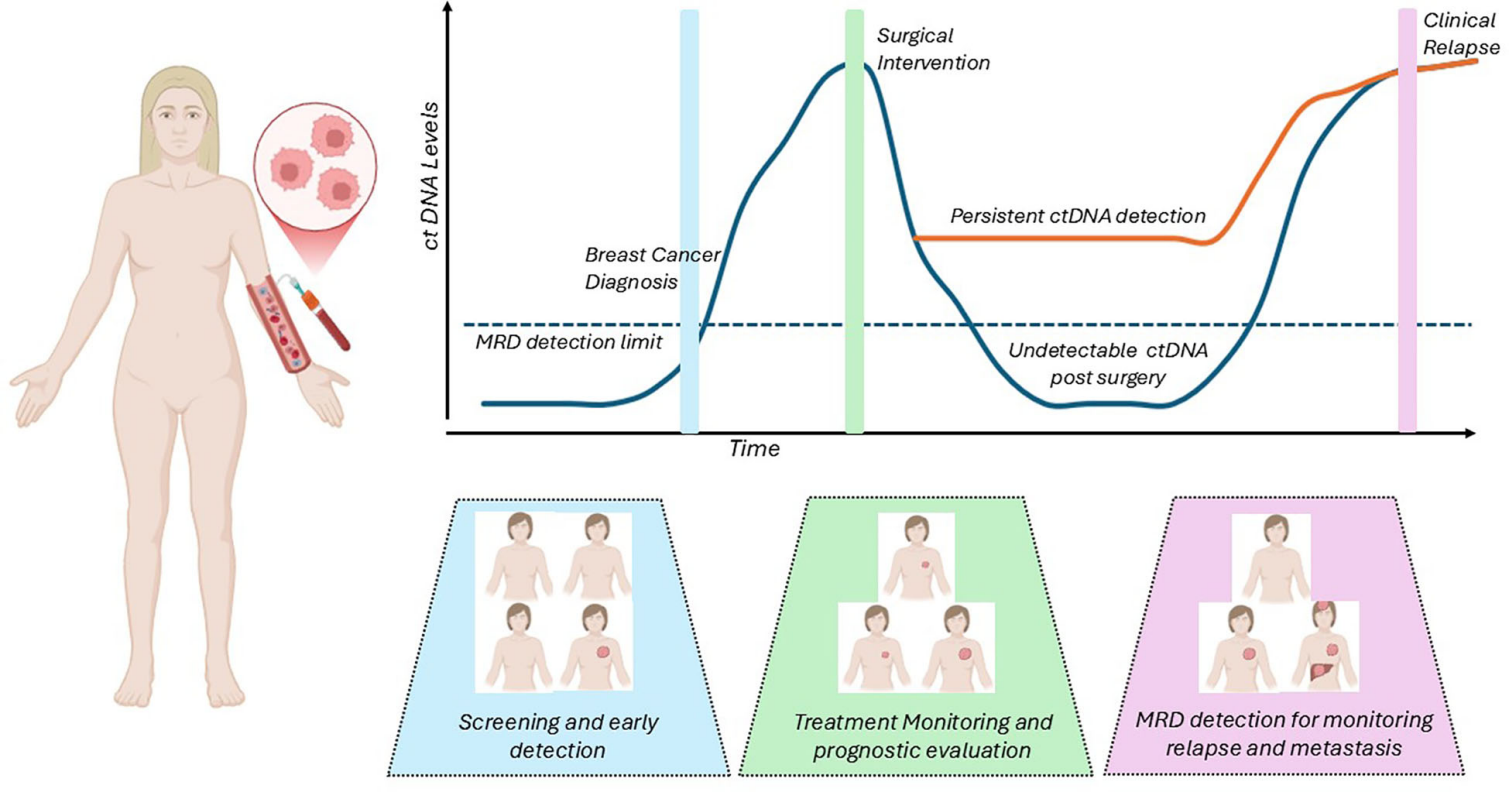
Potential applications of ctDNA monitoring in early breast cancer.

Assessment of circulating tumor DNA (ctDNA) is a tool in precision oncology that provides real time, non-invasive diagnostic testing that could complement screening techniques, augment treatment strategies and monitor for early recurrences (**Figure 1**). Variable shedding patterns in early stages of breast cancer (1) as well as in the different pathologic subtypes (2) have somewhat limited the use of ctDNA in early/non-metastatic disease. Advances in testing platforms have allowed for the development of ultrasensitive tests that detect DNA down to levels as low as 1 part per million, and their application in clinical management of early breast cancer is being explored in multiple large-scale, prospective studies. Several assays, especially tumor informed assays, have shown high rates of sensitivity and specificity in the detection of ctDNA, but to date there is no gold standard assay or direct comparison between the available options. ctDNA monitoring has been integrated into the management of lung cancer (3), and has made significant progress in guiding colon cancer therapy (4, 5), and though it remains a promising option, it is not widely used or a guideline recommended adjunct to treatment in breast medical oncology.

Several retrospective analyses of clinical trials and cohorts have shown that the monitoring of ctDNA can predict relapses in breast cancer, with a lead time of up to 3 years [ranging from 0–38 months] (6). The detection of ctDNA in the absence of overt metastases or recurrent disease indicates molecular evidence of cancer, defined as minimal residual disease (MRD). Detection of MRD via ctDNA before overt metastases may offer a unique opportunity to intervene, improving morbidity and outcome in this patient cohort. The ctDNA assays can be tumor-informed, where blood samples are tested for the presence of tumor specific mutations selected by sequencing the primary tumor making this panel patient specific, or they can be tumor-agnostic assays that are typically looking for cancer driver mutations, copy number aberrations, or cancer-derived methylation signals which are not patient specific but are cancer specific.

Here, we will review available data supporting the utility of ctDNA in non-metastatic breast cancer with a focus on its role in monitoring minimal disease or MRD, review prospective trials that are evaluating the integration of ctDNA surveillance into clinical practice, and MRD technology in development.

## ctDNA for early detection and screening

Monitoring ctDNA for minimal disease has potential applications throughout the spectrum of early-stage breast cancer, including in screening. While we have established and effective early detection modalities in breast cancer including screening mammograms, the integration of non-invasive ctDNA detection could be a valuable addition to the screening paradigm to increase sensitivity of current test results.

The multi-cancer early detection [MCED] test Galleri® is a commercially available ctDNA-based screening test that was validated following results from the Circulating Cell-Free Genome Atlas study that reported an excellent 99.5% specificity and 51.5% sensitivity. However, the sensitivity was lower at 30.5% in detecting breast cancer, with only 2.6% of Stage 1 cancers being detected with this assay [compared to over 90% in Stage 4 cancers] (7). In the prospective PATHFINDER trial, which screened over 6,600 asymptomatic participants with Galleri®, it was noted that while overall detection rates were acceptable, the sensitivity varied based on the type of malignancy and was lower in early stages (8). The 5 cases of breast cancer that were detected in this cohort were recurrences, and several missed cases were later diagnosed with conventional screening tests underscoring the limitation of this test in isolation.

CancerSEEK is another MCED test that integrates a ctDNA targeted mutation panel and multiplex protein profiling to identify malignancies. Although not commercially available at the time of this review, initial observational studies reported a median sensitivity of 70% across eight tumor types including breast cancer, with over 99% specificity (9). The DETECT study is a large-scale prospective trial enrolling 10,000 women to evaluate the combined utility of CancerSEEK and PET/CT imaging in identifying early-stage cancers (10).

Ongoing trials such as STRIVE, RENOVATE (11) and the NHS-Galleri trial (12) will further assess the clinical utility of these tests and guide future research initiatives in enhanced cancer screening. While MCED tests hold significant potential, the role of ctDNA in breast cancer screening remains complementary to established modalities. Continued innovation in assay sensitivity and large-scale validation studies will be critical to realizing its full potential in early detection.

## ctDNA in monitoring treatment response

Following a breast cancer diagnosis, ctDNA analysis is being actively investigated as a tool to assess therapeutic response and to guide escalation and de-escalation treatment decisions (13). Multiple studies have demonstrated that changes in ctDNA levels correlate with pathological response, recurrence-free survival [RFS], and overall survival [OS] (2, 14–17).

In the I-SPY2 trial, patients with HER2-negative early breast cancer were monitored with ctDNA at multiple timepoints during neoadjuvant chemotherapy [NAC]. Persistent ctDNA positivity via a Signatera™ assay at three weeks after NAC completion was significantly associated with a lack of pathologic complete response [pCR] (82% vs 52% non-pCR; odds ratio [OR] 4.33, *P* = 0.012), while early ctDNA clearance predicted improved outcomes in terms of pCR rates in triple-negative breast cancer [TNBC] patients (*P* = 0.0002) (18). Similar findings were reported by Cavallone et al., who demonstrated that a significant decline in variant allele frequency [VAF] after one cycle of NAC predicted pCR in TNBC patients (15). Conversely, persistent or rising ctDNA levels at the end of treatment were associated with poorer RFS (hazard ratio [HR] = 0.29 (95% confidence interval [CI] 0.08–0.98), *P* = 0.046), and OS (HR = 0.27; 95% CI 0.075–0.96, *P* = 0.043) (15). In the Translational Breast Cancer Research Consortium [TBCRC]-030 trial, post-treatment ctDNA clearance via a tumor-informed assay strongly correlated with favorable residual cancer burden [RCB] scores, and an impressive 285-fold decrease was noted in the ctDNA tumor fraction in the responders group after 3 weeks of treatment (2). Additionally, recent results from the PREDICT-DNA/TBCRC 040 trial showed TNBC patients with detectable ctDNA prior to surgery were approximately 12 times more likely to experience a recurrence regardless of pCR (HR = 12.8; 95% CI 2.3-71.5). While undetectable ctDNA after NAC did not reliably predict pCR, it was associated with improved prognosis (19).

Other studies have corroborated that ctDNA dynamics during NAC can help risk stratify patients beyond RCB classification. For example, the DAPHNe trial, which assessed the NeXT Personal^®^ ctDNA assay in HER2-positive patients, showed that patients with undetectable ctDNA following NAC had excellent long-term outcomes, even when residual disease was present at surgery. Among the 50 patients evaluated, 92% patients had detectable ctDNA at baseline, with a reduction to only 4% of patients with detectable ctDNA after 12 weeks of NAC. After a median follow up of 50 months, none of the 50 patients had recurred including the 34% patients that had residual disease at surgery (20). In the aforementioned I-SPY2 trial, ctDNA negativity after NAC showed a significant association with improved distant recurrence-free survival [DRFS] irrespective of RCB status at surgery (*P <*0.0001). And in patients with hormone receptor-positive, HER2-negative [HR+/HER2-] breast cancer and RCB II/III status, decreased risk of metastatic recurrence was reported in patients with a negative ctDNA result prior to surgery when compared to patients with positive result (HR 5.65; 95% CI 2.45–12.99) (16).

Collectively, these data indicate that ctDNA assessment after neoadjuvant therapy may offer a more accurate prognosis than standard clinicopathologic markers, such as RCB. We also surmise from these trials that, if validated in large intervention trials with ultrasensitive levels of detection, strategies for treatment de-escalation could be safely considered in patients with undetectable ctDNA after NAC given their excellent prognosis (5, 21), or treatment escalation if ctDNA identified due to poorer anticipated prognosis.

## ctDNA for MRD surveillance and recurrence prediction

In the adjuvant setting, ctDNA MRD monitoring has been assessed for early identification of distant disease spread. MRD, defined as the presence of ctDNA in the absence of radiographic disease, has been consistently associated with a heightened risk of clinical relapse. Several assays are currently used for MRD detection, including tumor-informed platforms [e.g., Signatera™, RaDaR®, NeXT Personal^®^, and Invitae Personalized Cancer Monitoring™] and tumor-agnostic platforms [e.g., Guardant Reveal™].

Signatera™, a tumor-informed assay validated in multiple studies, has demonstrated 85-90% sensitivity in detecting MRD in early-stage breast cancer (22). In the Exploratory Breast Lead Interval Study [EBLIS] study, ctDNA was detected ahead of overt recurrence in 30 of 34 patients that relapsed, with a lead time of up to 38 months [median 10.5 months] (6). Natera is currently developing next-generation MRD tests, including a tissue-agnostic assay and an ultra-sensitive test that will detect ctDNA down to single-digit parts per million. With these ultra-sensitive tests, the detection thresholds are often extrapolated from low levels of ctDNA input, and variability in the precision of the result is possible (23).

NeXT Personal^®^, a tumor-informed platform capable of detecting ultra-low levels of ctDNA (detection threshold of 1 part per million), demonstrated 100% sensitivity and specificity for MRD detection in the ChemoNEAR study (24). At a median follow-up of 76 months, detection of ctDNA was associated with an increased risk of relapse (HR undefined, *P <*0.0001), decreased OS (*P <*0.0001) and a median lead time of 12.5 months. Similarly, the Invitae Personalized Cancer Monitoring™ assay achieved 76.9% sensitivity and 100% specificity, with a median lead time of nearly one year, in a 61 patient cohort with high risk breast cancer who underwent longitudinal ctDNA monitoring (25).

Tumor-agnostic assays, such as Guardant Reveal™, are generally considered to be less sensitive for the detection of ctDNA MRD since they evaluate standard mutation panels that may not be present in a given patient’s tumor, though may offer a more timely and cost effective approach. Guardant Reveal™ detects ctDNA by analyzing methylated regions of DNA optimized to detect breast cancer DNA from non-cancerous cell-free DNA. In early-stage TNBC, Guardant Reveal™ demonstrated a 71% sensitivity for detecting distant recurrence, with a median lead time of 5 months (26). In another analysis of the Guardant Reveal™ test run on the Infinity platform in 83 patients with early breast cancer, ctDNA positivity was strongly corelated with risk of recurrence (HR=7.02; 95% CI 1.82-27.2, *P* = 0.001) (27).

Although these assays have demonstrated the ability to identify those at highest likelihood of recurrence and can detect disease well before clinical relapses, MRD detection alone cannot improve outcomes without accompanying therapeutic intervention.

## Therapeutic intervention trials: the path forward for incorporation of ctDNA detection into clinical practice

Beyond risk stratification, the integration of ctDNA MRD monitoring into clinical practice hinges on confirmation of its utility in changing clinical outcomes, i.e. does early treatment guided by ctDNA detection lead to improved survival or reduced recurrence.

The c-TRAK TN trial was the first trial to prospectively explore ctDNA-guided therapy. This phase II trial enrolled patients with early-stage TNBC who had completed surgery and adjuvant therapy. Those with detectable ctDNA were randomized to pembrolizumab or observation. However, due to frequent radiologic evidence of metastatic disease at the time of ctDNA detection, only a few patients received intervention, and the trial was underpowered to determine clinical benefit (28).

Similarly, the ZEST trial aimed to assess whether niraparib could improve invasive disease-free survival [iDFS] in *BRCA1/2*-mutated, HER2-negative breast cancer patients with MRD positivity. Despite screening over 2,700 patients, only 18 were ultimately randomized before the trial was terminated early due to low accrual and high rates of concurrent radiographic recurrence (29).

The DARE trial investigates whether a therapeutic switch to palbociclib and fulvestrant in high-risk HR+/HER2– breast cancer patients with ctDNA-detected MRD can delay clinical recurrence. Interim results presented at the American Society of Clinical Oncology [ASCO] 2025 Annual Meeting showed that among the 507 patients who were screened, 99.5% with sustained ctDNA negativity remained recurrence-free at 27.4 months. Among 60 ctDNA-positive patients, 73% had no detectable disease on imaging, and at time of data review 38 patients had been randomized. Those receiving escalated treatment experienced a twofold higher ctDNA clearance rate at 3 months. Longer-term follow-up will inform whether this strategy translates into decreased clinically detected recurrence or improved survival (30).

As we learn from these challenges, hope remains for outcome results with other ongoing MRD intervention trials including LEADER, TRAK-ER and TREAT ctDNA among others (**Table 1**). In parallel, de-escalation trials are exploring whether therapy can be safely minimized in MRD-negative patients. For example, the SAFE-DE trial is evaluating post-operative MRD in patients with early stage, lower risk HER2-positive breast cancer and TNBC. While treating providers will not be mandated to provide certain treatments based on ctDNA results, treatment choices will be tracked based on the ctDNA result [i.e. will chemotherapy be deferred if MRD negative, or a regimen escalated if MRD positive] (31). As data matures, these studies will inform whether ctDNA-guided treatment adjustments can be incorporated into standard care, and what degree of assay accuracy is required for safe decision-making.

**Table 1:** Prospective interventional clinical studies utilizing ctDNA for MRD monitoring.

Trial name	NCT ID	Patient inclusion	Number of patients enrolled or planned	ctDNA analysis test used	Study design
DARE	NCT04567420	Stage II-III ER+/HER2- on adjuvant endocrine therapy	540	Signatera™ (tumor-informed)	ctDNA+ patients randomized to: (1) continue standard of care (SOC) endocrine therapy, or (2) escalate to palbociclib + fulvestrant
LEADER	NCT03285412	T1c-T4c, any N, ER+/HER2- on adjuvant endocrine therapy	120	Signatera™ (tumor-informed)	ctDNA+ patients randomized to: (1) continue SOC endocrine therapy, or (2) escalate to ribociclib + endocrine therapy
TRAK-ER	NCT04985266	High-risk ER+/HER2- on adjuvant endocrine therapy	1100	RaDaR™ (tumor-informed)	ctDNA+ patients randomized to: (1) continue SOC endocrine therapy, or (2) escalate to palbociclib + fulvestrant for up to 24 months
TREAT	NCT05512364	High-risk ER+/HER2- on adjuvant endocrine therapy	120	RaDaR™ (tumor-informed)	ctDNA+ patients randomized to: (1) continue SOC endocrine therapy, or (2) switch to elacestrant
SAFE-DE	NCT05058183	Stage 1 breast cancer that is HER2+ or triple negative	400	Signatera™ (tumor-informed)	Adjuvant treatment choices (escalation or de-escalation) will be analyzed, incorporating post-operative ctDNA result
ASPIRA	NCT04434040	Triple negative breast cancer, with residual disease following neoadjuvant chemotherapy	40	Unknown	Patients that are ctDNA+ will be enrolled for serial monitoring of ctDNA during therapy with atezolizumab and sacituzumab govitecan
PERSEVERE	NCT04849364	Triple negative breast cancer, with residual disease following neoadjuvant chemotherapy	197	Unknown	3 arm study: (1) ctDNA+ patients with actionable targets will receive genomically-directed therapy; (2) ctDNA+ patients without actionable genomic targets and (3) ctDNA- patients will receive capecitabine and pembrolizumab, or treatment of physician’s choice

## Future technology for assessing MRD in breast cancer

Many questions remain and the breadth of potential application of MRD evaluation in early breast cancer is expansive. Similar to its application in other malignancies, for ctDNA testing to be widely utilized the developing tests should inform therapeutic interventions that decrease the overall financial burden associated with cancer care (32), and the sensitivity and specificity of the test needs to be high. Or, what error would be allowable for patients and treating providers when making treatment decisions. Current blood-based measures of breast cancer disease activity [e.g. tumor markers] have unacceptably low levels of sensitivity and specificity for clinical decision-making, with a reported sensitivity for CEA of 88% (33), and even lower for CA15–3 and CA27-29 (34). Increased sensitivity has been achieved with blood genomic evaluations, with the highest specificity and sensitivity achieved with more comprehensive evaluations. In one comparison of whole genome sequencing [WGS] versus whole exome sequencing [WES], the WGS assay achieved 100% sensitivity, compared to 84% with the WES assay (25).

Beyond WGS, quantification of somatic structural variants [SV, including breakpoints and rearrangements] and copy number aberrations may allow for ultrasensitive MRD tracking. Structural variants often correlate with driver alterations but arise from breakpoints unique to each tumor. This specificity to the tumor minimizes false positives that can occur from clonal hematopoiesis of indeterminate potential [CHIP] or next generation sequencing [NGS] errors. Elliot et al. serially assessed tumor-specific SVs via digital PCR in 100 patients receiving NAC for breast cancer [all subtypes], with over 2500 breakpoints identified. ctDNA was detected in 96% (91/95) of participants at baseline with a median VAF of 0.15% (range: 0.0011%–38.7%). ctDNA via SV post-operatively was 100% sensitive for predicting distant recurrence, with a median lead time of 417 days (range 4–1,931 days) (35). Copy number analysis via ultra-low-pass WGS [ULP-WGS] offers the most cost-effective method for detecting ctDNA. In one study in breast cancer, patients with detectable ctDNA by ULP-WGS at the completion of NAC had a higher risk of recurrence than those with undetectable ctDNA, with a 2-year event free survival of 40% compared to 83.9% if ctDNA negative (36).

Measuring epigenetic changes in cancer – such as methylation – is another promising frontier for evaluation of deep MRD. Dysregulated DNA methylation is a universal feature of cancer, and patterns can change in response to therapies and tumor biology (37). In one study of 7 patients with triple positive breast cancer, a genome-wide analysis of ctDNA methylation identified distinct patterns of methylation in patients achieving a pCR, which were not appreciated in patients with residual disease (38). These changes may represent some of the earliest signals of cancer cell activity. As aforementioned, GRAIL has already commercialized the DNA methylation-based Galleri^®^ test for early detection of cancer, attempting to identify cancerous methylation signals that highlight a risk of cancer cells prior to a cancer diagnosis. Guardant has also added methylation testing to its platform. GuardantReveal™ and GuardantINFINITY™ detect MRD via genomic alterations and an assessment of differential methylation. With the GuardantINFINITY™ test, the methylation score was able to accurately identify 95% of patients with breast cancer [versus healthy controls] and performed better than tumor-agnostic genomic profiling to quantify ctDNA (39, 40).

Fragmentomics - the study of circulating cell-free DNA (ccfDNA) fragmentation patterns – may also have potential for use in oncological assays, with increasing sensitivity. Mouliere et al. (41)assessed 200 cancer patients for the presence of shorter ctDNA fragments [between 90–150], with improved detection of ctDNA and clinically actionable mutations and copy number alterations when incorporating fragment size analysis. Additionally, Cristiano et al. (42)were able to discriminate between 215 healthy individuals from 208 cancer patients (AUC = 0.94) using a machine learning model trained on ccfDNA fragment size and coverage across the genome, demonstrating the potential for ctDNA fragment patterns to be used as cancer biomarkers. Soon, we may be able to combine these approaches – assessment of DNA methylation and fragmentomics – for improved ctDNA detection (43).

## Conclusion

While ctDNA evaluation in early breast cancer has been clearly linked with prognosis, we continue to await reliable data that demonstrates acting on MRD improves patient outcomes. Ongoing prospective studies will be pivotal in establishing the clinical utility of ctDNA MRD surveillance and its role in precision treatment strategies. Efforts to develop standardized test assays that can detect ultralow levels of ctDNA are also needed, allowing for the most reliable MRD testing to inform clinical decisions without compromising outcomes. Ultimately, the pace of discovery has been encouraging, and it is anticipated integration of ctDNA in the management of non-metastatic breast cancer may be a reality in the near future.
